# Inhibition of β-catenin/p300 interaction proximalizes mouse embryonic lung epithelium

**DOI:** 10.1186/s40247-014-0008-1

**Published:** 2014-09-11

**Authors:** Tomoyo Sasaki, Michael Kahn

**Affiliations:** Department of Biochemistry and Molecular Biology, Keck School of Medicine, University of Southern California, 1450 Biggy Street, Los Angeles, CA 90033 USA; Center for Molecular Pathways and Drug Discovery, Keck School of Medicine, University of Southern California, 1450 Biggy Street, Los Angeles, CA 90033 USA; Norris Comprehensive Cancer Center, Keck School of Medicine, University of Southern California, 1450 Biggy Street, Los Angeles, CA 90033 USA

**Keywords:** Lung, Branching, Differentiation, Wnt, β-catenin, p300, CBP, IQ1, ICG-001, Small molecule inhibitor

## Abstract

**Background:**

Wnt/β-catenin signaling has been suggested to regulate proximal-distal determination of embryonic lung epithelium based upon genetically modified mouse models. The previously identified and characterized small molecule inhibitor IQ1 can pharmacologically decrease the interaction between β-catenin and its transcriptional coactivator p300, thereby enhancing the β-catenin/CBP interaction. Inhibition of the β-catenin/p300 interaction by IQ1 blocks the differentiation of embryonic stem cells and epicardial progenitor cells; however, whether differential coactivator usage by β-catenin plays a role in proximal-distal determination of lung epithelium is unknown.

**Methods:**

We examined the effects of inhibiting the β-catenin/p300 interaction with IQ1 on lung branching morphogenesis in mouse embryos in utero and mouse embryonic lung organ culture ex vivo. The phenotype of IQ1 treated lungs was analyzed by epithelial staining, histology, quantitative PCR and in situ hybridization.

**Results:**

Inhibition of the β-catenin/p300 interaction by IQ1 disrupted the distal branching of mouse lung epithelium both in utero and ex vivo. IQ1 proximalized lung epithelium with decreased expression of the genes Bmp4 and Fgf10, hallmarks of distal lung determination, and increased expression of the proximal genes Sox2 and Scgb1a1 (CC10) as shown by quantitative PCR and in situ hybridization. The disruption of branching was reversible ex vivo as branching was reinitiated after removal of IQ1 from the media.

**Conclusions:**

The results demonstrate that the β-catenin/p300 interaction plays a critical role in proximal-distal determination of the epithelium in mouse lung branching morphogenesis and β-catenin/p300 inhibition pharmacologically proximalizes lung epithelium.

## Background

The ability to regulate proliferation and differentiation of the lung has important consequences in regards to both premature birth and bronchopulmonary dysplasia [1] and diseases of the adult lung i.e. idiopathic pulmonary fibrosis [2]. Furthermore, within the context of regenerative medicine, the ability to control lung development may replace conventional lung transplantation, which has inevitable problems associated with both a shortage of suitable donors and immunogenicity leading to the requirement for long term immunosuppression and fibrotic complications [3]. Regeneration is in essence a recapitulation of development. Therefore, understanding the signaling pathways and decision points that control lung development is a critical goal. Lung development involves branching morphogenesis. The primordial epithelial tube undergoes repetitive branching at distal tips resulting in a tree-like structure. Lung branching morphogenesis is regulated by multiple signaling pathways including the FGF pathway [4] and the Wnt signaling pathway [5]. β-catenin is the central signaling mediator of canonical Wnt signaling. Therefore, β-catenin has repeatedly been genetically deleted for loss-of-function analysis or stabilized for gain-of-function analysis in mice to study Wnt signaling. During lung development, tissue-specific deletion of β-catenin in the epithelium was found to proximalize the epithelium by expanding the proximal airway and inhibiting distal airway growth [6]. Based on this analysis, β-catenin was suggested to regulate proximal-distal determination of the epithelium during lung morphogenesis. However, beyond its role as a transcriptional activator of Wnt signaling, β-catenin is also an important protein component of adherens junction, which is critically responsible for epithelial cell-cell adhesion. Thus, genetic deletion of β-catenin in mice results in both disruption in cell-cell adhesion and the transcriptional role of β-catenin, making analysis of the phenotype complex [5].

Chemical genetic approaches offer a complementary method to genetic manipulation, to selectively modulate the transcriptional role of β-catenin. To activate transcription, β-catenin must translocate to the nucleus to interact with transcription factors e.g. members of the TCF/LEF in canonical Wnt signaling as well as various transcriptional co-activators. We have identified and characterized a number of small molecules that specifically inhibit the protein-protein interaction between β-catenin and either of its two Kat3 coactivators i.e. Creb binding protein (CBP) or p300. Specifically inhibiting either the β-catenin/CBP or the β-catenin/p300 interaction does not disturb other protein-protein interactions involving β-catenin, including the β-catenin/E-Cadherin interaction that is critically responsible for epithelial cell-cell adhesion. Therefore, specific well-characterized small molecule pharmacologic tools provide an advantage for loss-of-function analysis of multifunctional proteins like β-catenin. An additional potential advantage of utilizing a pharmacologic approach is the ability to temporally utilize small molecule pharmacologic agents to manipulate cell proliferation and differentiation for the purpose of regenerative medicine. IQ1 (C_21_H_22_N_4_O_2_, molecular weight 362.42) and ICG-001 (C_33_H_32_N_4_O_4_, molecular weight 548.63) are specific inhibitors of the interaction between β-catenin and the transcriptional coactivators p300 and CBP respectively. IQ1 inhibits the interaction between β-catenin and p300 indirectly via targeting the PR72/130 subunit of PP2A and thereby blocking PP2A/Nkd complex formation [7]. On the other hand, ICG-001 directly inhibits the interaction between β-catenin and CBP via binding to the amino terminus of CBP [8]. Using these small molecules, we have previously demonstrated the distinct roles that the two coactivators p300 and CBP in β-catenin dependent transcriptional gene regulation, despite their high degree of identity and even higher homology. Inhibition of the β-catenin/p300 interaction by IQ1 prevented the differentiation of embryonic stem cells [7], whereas inhibition of β-catenin/CBP interaction enhanced the differentiation of neural progenitor cells [9] and human embryonic stem cells (hES cells) [10]. However, the role of β-catenin differential coactivator utilization in lung development and branching morphogenesis is unknown.

To investigate the role of β-catenin differential coactivator utilization in murine lung development and branching morphogenesis, we treated mouse embryonic lungs with IQ1 *in utero* and *ex vivo* to selectively inhibit the β-catenin/p300 interaction and analyzed the phenotype by epithelial staining, histology, quantitative PCR and *in situ* hybridization.

## Methods

### Compounds

IQ1 and ICG-001 were synthesized in our laboratory as previously described [7,11]. IQ1 and ICG-001 were dissolved in dimethyl sulfoxide (DMSO) to prepare 100 mM or 500 mM stock solutions. Equal volumes of DMSO were used for controls.

### Mice

TOPGAL mice were obtained from The Jackson Laboratory. Vaginal plug at noon was considered as day 0.5 of pregnancy (E0.5). For administration of IQ1 or ICG-001 to embryos *in utero*, timed pregnant mice were fed IQ1 (72 or 144 mg/kg/day) or ICG-001 (220 mg/kg/day) mixed in peanut butter in addition to their regular diet. The animal study protocol was approved by the IACUC at the University of Southern California.

### Whole mount NBT/BCIP and β-galactosidase staining

Lung samples were fixed in 4% paraformaldehyde for 1 hour to overnight and washed in phosphate buffered saline (PBS). The lungs were incubated in a solution of NBT/BCIP Ready-to-Use Tablets (Roche Applied Science) according to the manufacturer’s directions. After development of color, excess staining was removed by dehydration and rehydration through a series of methanol washes. The stained lungs were cleared in graded steps with glycerol before photographing. Branching tips of the stained lungs were counted under a bright-field stereomicroscope. For histological analysis, the stained lungs were re-fixed in 4% paraformaldehyde, embedded in paraffin, sectioned and counterstained with Nuclear Fast Red (Electron Microscopy Sciences). For β-galactosidase staining, lungs were dissected, fixed in 0.5% glutaraldehyde with 2 mM of magnesium chloride and stained as described previously. The images were captured using an AxioImager Z1 and AxioVision digital image processing software (Carl Zeiss) or SZX7 stereomicroscope and infinity2 imaging system (Olympus).

### Lung organ culture

Lungs were dissected from mouse embryos in cold sterile Hanks’ Balanced Salt Solution under a stereomicroscope. The lungs were randomized by size and obvious outliers were excluded. The lungs were placed on a polycarbonate membrane filter (Whatman) floating on 1 ml of serum-free BGJb medium (Invitrogen) with penicillin/streptomycin in 60 mm Center-well Organ Culture Dishes (BD Falcon). The lung explants were positioned at the gas–liquid interface on the membrane filters. The Cultures were kept at 37°C in 5% CO2 and media was changed daily.

### Quantitative PCR (qPCR)

The lung explants were homogenized with TRIzol® reagent (Invitrogen) by passing through a 25-gage needle and total RNA was isolated according to the manufacturer’s instructions. The concentration of total RNA was measured using a NanoDrop® spectrophotometer and reverse transcription was performed with an iScript cDNA Synthesis Kit (Bio-Rad). Quantitative PCR amplification was performed using SYBR Green PCR master mix reagent (BioRad) and a MyiQ thermal cycler (BioRad) with the following gene-specific primers: mouse *Bmp4* (CGTTACCTCAAGGGAGTCGAGATTG, TCTTATTCTTCTTCCTGGACCGCTG), mouse *Fgf10* (TGCACATACATGAGCCCTTTGT, TTTGCTCAGGTTAAGCCCCAG), mouse *Nkx2-1* (AAATTTGGGGGTCTTTCTGG, AGAGTGCATCCACAGGGAAG), mouse *Shh* (ACTCACCCCCAATTACAACCC, TGCTCCCGTGTTTTCCTCA), mouse *Scgb1a1* (CAGCTGAAGAGACTGGTGGAT, TGTTAGATTTTCTCCGTGAGCTT). Melt curve analysis and gel electrophoresis were used to examine the specificity of amplified products. Data were normalized to the reference gene, mouse *Gapdh*. Relative expression levels were calculated using the 2^-ddCt method. Data are presented as mean ± SD. Differences in means between two experimental groups were analyzed using two-sample, two-tailed Student’s t-test. *p* < 0.05 was considered significant.

### Whole mount *in situ* hybridization

The lung explants were fixed in 4% paraformaldehyde for 4 hours to overnight, stored in 100% methanol at −20°C, bleached in 3% hydrogen peroxide in methanol, rehydrated and subsequently underwent *in situ* hybridization as previously described. Digoxigenin-labeled anti-sense RNA probes were synthesized from subcloned mouse gene templates using DIG RNA Labeling Kit (Roche Applied Science). Hybridization was colorized with ALP conjugated anti-DIG antibody (Roche Applied Science) and NBT/BCIP Ready-to-Use Tablets (Roche Applied Science). Experiments were independently repeated at least three times. The plasmids including cDNA of mouse *Nkx2-1* and *Bmp4* were generously provided by Dr. Changgong Li.

### Histology and immunohistochemistry

Lung samples were fixed in 4% paraformaldehyde overnight, embedded in OCT compound or paraffin and sectioned. For immunohistochemistry, the sections were blocked in 1% bovine serum albumin (BSA) (Jackson ImmunoResearch) and incubated with primary antibodies. The antibodies used were FITC mouse anti-β-catenin (1:400, BD Transduction Laboratories) and anti-α-SMA (1:200, Sigma). For whole mount PECAM-1 staining, fixed lungs were dehydrated in methanol, bleached in 3% hydrogen peroxide in in methanol, rehydrated, blocked in 1% BSA, incubated with anti-PECAM-1 antibody (1:200, Santa Cruz, sc-1506) overnight at 4°C, washed in PBS, incubated with HRP-conjugated anti-goat IgG (Santa Cruz) and colorized with diaminobenzidine (DAB) substrate (Sigma).

## Results

### Alkaline phosphatase activity marks murine lung epithelium during branching morphogenesis

**Figure 1:**
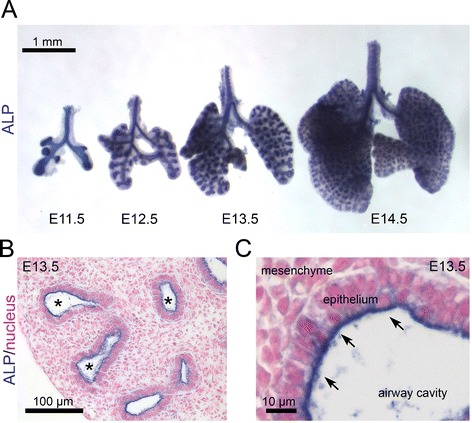
**Alkaline phosphatase (ALP) activity marks epithelial branching structures in embryonic mouse lung. (A)** Mouse embryonic lungs from four different embryonic stages i.e. E11.5, E12.5, E13.5 and E14.5 were stained with ALP substrate NBT/BCIP. Endogenous ALP activity in blue-purple visualizes epithelial branching morphogenesis in the whole-mount lungs. **(B)** Histology of the stained E13.5 lung shows the specific ALP activity around the airway cavities (*), but not in the mesenchyme. **(C)** Higher magnification shows that the ALP activity is specific to the apical surface of the lung epithelial cells (arrows).

Wnt signaling apparently plays a critical role during lung branching morphogenesis. We found that murine embryonic lung expresses endogenous alkaline phosphatase (ALP) activity in the epithelium. The epithelium can be selectively stained utilizing the endogenous ALP activity with the chromogenic substrate, nitro-blue tetrazolium chloride and 5-bromo-4-chloro-3′-indolyphosphate p-toluidine salt (NBT/BCIP). We therefore sought to utilize NBT/BCIP to visualize epithelial branching morphogenesis in whole mount mouse lung. In the event, we stained mouse lung at four different embryonic stages from E11.5 to E14.5 with NBT/BCIP. We observed blue-purple staining specifically visualizing lung epithelial structures during branching morphogenesis at all four time points (Figure 1A). Next, we sectioned the stained lungs for histological analysis and confirmed that the blue-purple staining was localized on the apical surface of the lung epithelium and not in the mesenchyme (Figure 1B and 1C). These results suggest that endogenous ALP activity is a specific marker for embryonic mouse lung epithelium and NBT/BCIP, chromogenic substrate of ALP, can be used to visualize the epithelial branching structure in whole mount mouse lung using bright-field microscopy.

### The β-catenin/p300 interaction during lung branching morphogenesis

**Figure 2:**
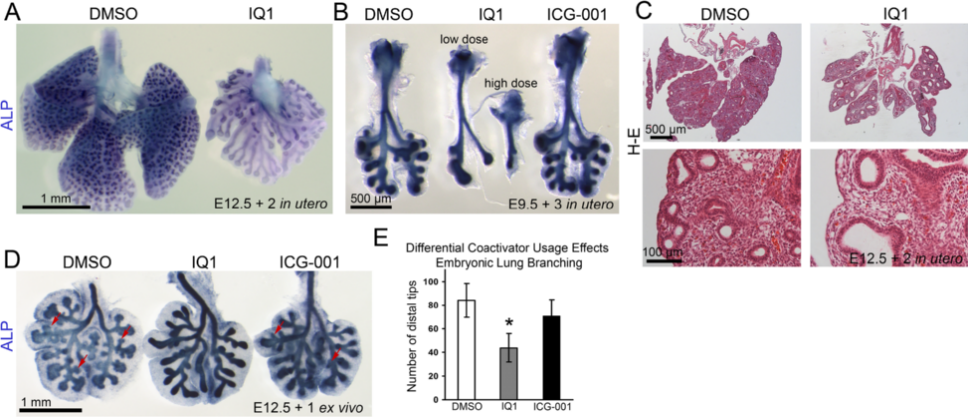
**Inhibition of the β-catenin/p300 interaction disrupts branching morphogenesis in mouse lung. (A)** Lungs from embryonic mice treated with the β-catenin/p300 inhibiter IQ1 (right) or DMSO control (left) *in utero*. Pregnant mice received IQ1 (72 mg/kg/day) orally to expose the embryos to IQ1 *in utero* from E12.5 for 2 days (E12.5 + 2). The lungs of the embryos were dissected and the epithelium was stained with the ALP substrate NBT/BCIP. IQ1 decreased the size of the lung but elongated the airways. **(B)** Pregnant mice received IQ1 (72 mg/kg/day for low dose, 144 mg/kg/day for high dose) or ICG-001 (220 mg/kg/day) orally to expose the embryos *in utero* from E9.5 for 3 days (E9.5 + 3). IQ1 inhibited lung branching morphogenesis but ICG-001 did not. **(C)** H-E staining shows that IQ1 dilated the airways and decreased the density of mesenchymal cells. **(D)** E12.5 mouse embryonic lungs were cultured with either DMSO, IQ1 (10 μM) or ICG-001 (10 μM) for 24 hours and stained with ALP substrate NBT/BCIP. The arrows indicate new distal tips in DMSO and ICG-001 treated explants. IQ1 disrupted branching morphogenesis *ex vivo*. **(E)** IQ1 significantly decreased the number of the distal tips after 24 hour culture of E12.5 lungs. Data are mean ± SD from five lungs per group. *p < 0.025 vs. control by t-test.

**Figure 3:**
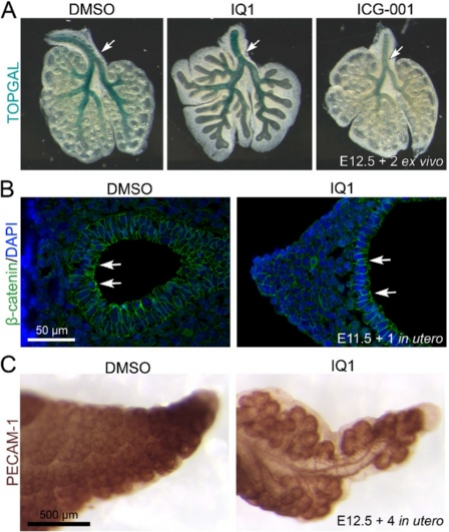
**Histological analyses of IQ1 treated lungs. (A)** TOPGAL, the Wnt signaling reporter mouse shows activation of β-catenin/LEF/TCF reporter by expression of β-galactosidase. E12.5 lung explants of TOPGAL mouse were treated with either DMSO (control), IQ1 (10 μM) or ICG-001 (10 μM) for 48 hours. β-galactosidase (blue) activity is observed in the bronchial epithelium of the proximal airway (arrows). IQ1 did not decrease β-galactosidase activity, whereas ICG-001, a known CBP/catenin antagonist, decreased β-galactosidase activity in the proximal airway. **(B)** Immunohistochemistry shows localization of β-catenin at the cell membrane of epithelial cell (arrows) and mesenchymal cells. **(C)** Whole mount PECAM-1 staining (brown) shows capillaries around the distal tips. Four days of IQ1 treatment *in utero* did not significantly disrupt formation of capillaries as judged by PECAM staining.

To investigate the role of differential β-catenin coactivator usage during lung branching morphogenesis, we used the coactivator selective small molecule inhibitors of the β-catenin/p300 [7] and β-catenin/CBP [8] interaction that we previously identified and have utilized extensively [2,7,9,12]. To inhibit the β-catenin/p300 interaction during lung morphogenesis, we treated E12.5 mouse embryos with IQ1 *in utero* for two days via oral administration of IQ1 to their pregnant mothers. In the embryonic mice at day E14.5, we found that the inhibition of the β-catenin/p300 interaction disrupted lung branching morphogenesis. IQ1 decreased the size of the lung and dramatically decreased the number of branching tips (Figure 2A). Despite the branching inhibition at the distal tips, IQ1 caused an elongation of the proximal airway compared to the control lung at E14.5 (Figure 2A). This elongation of the proximal airways suggests that the impact of IQ1 is not due simply to growth inhibition. IQ1 also inhibited branching with 3 day treatment initiating at E9.9; however, ICG-001 did not affect branching *in utero* (Figure 2B). Further, we observed that IQ1 caused airway dilation and decreased the density of mesenchymal cells (Figure 2C). Next, we applied IQ1 to lung organ culture to confirm that the disruption of branching *in utero* did not result from overall poor general condition of the embryos, as we have previously demonstrated that IQ1 also affects cardiac and vascular system development [13]. We cultured E12.5 lung explants at a gas–liquid interface on membrane filters for 24 hours. We examined the effects of both the specific small molecule β-catenin/CBP inhibitor ICG-001, as well as the β-catenin/p300 inhibitor IQ1 in this model. We found that inhibition of the β-catenin/p300 interaction with IQ1, but not the β-catenin/CBP interaction with ICG-001, significantly decreased the number of distal tips at 24 hours *ex vivo* (Figure 2D and 2E). The differences are even more striking after two days of culture (Figure 3A). ICG-001 slightly slowed the growth of explants, however we observed new formation of distal tips in ICG-001 treated but not in IQ1 treated cultures (Figure 2D and 3A). The effects of differential coactivator modulation in lung organ culture confirmed that the disruption of branching morphogenesis *in utero* was a direct effect on the lung and did not result from overall inhibition of embryonic development. Our results suggest that the β-catenin/p300 interaction plays a critical role during mouse lung branching morphogenesis.

### Inhibition of β-catenin/p300 interaction proximalizes the lung epithelium

**Figure 4:**
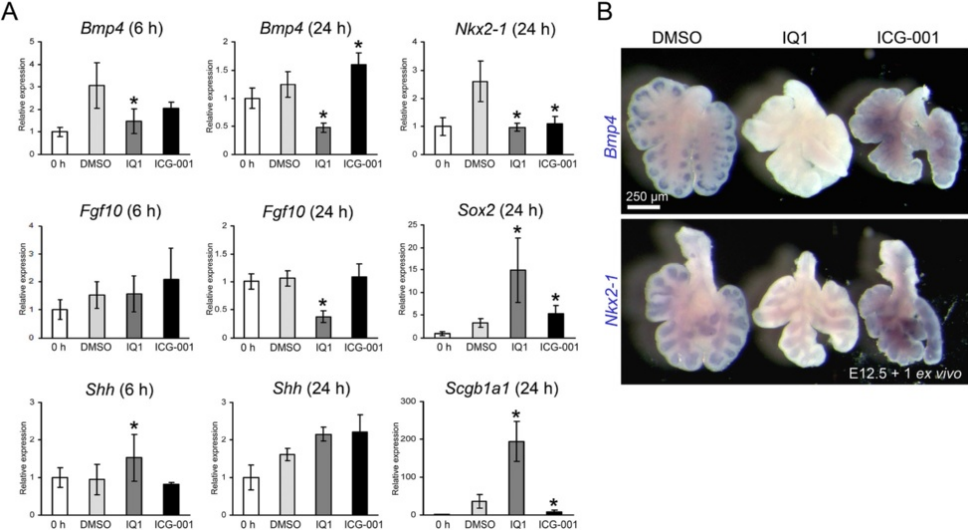
**Gene expression analyses of IQ1 treated lung explants. (A)** Quantitative PCR (qPCR) analysis of the lung explants. Lung explants were cultured with either DMSO (control), IQ1 (10 μM) or ICG-001 (10 μM) for 6 hours or 24 hours. Relative expression changes compared to E12.5 lungs without culture (0 h) are presented. IQ1 decreased the expression of *Bmp4* significantly at 6 hours and 24 hours. IQ1 increased the expression of *Sox2* and *Scgb1a1*. Explants groups: 6 hours treatment (0 h, *n* = 8, DMSO *n* = 8, IQ1, *n* = 9, ICG-001, *n* = 3), 24 hours treatment (0 h, *n* = 6, DMSO, *n* = 6, IQ1, *n* = 6, ICG-001, *n* = 6). Data are presented as mean ± SD. *p < 0.05 vs. control by t-test. **(B)** Whole mount *in situ* hybridization shows local expression of *Bmp4* and *Nkx2-1* in the distal epithelium. IQ1 decreased the expression of *Bmp4* and *Nkx2-1* at 24 hours.

The elongated proximal airways observed in the IQ1 treated lungs led us to investigate the hypothesis that specific inhibition of the β-catenin/p300 interaction proximalizes the lung epithelium. To test this hypothesis, we analyzed the expression of both distal and proximal genes in lung explants by quantitative PCR (qPCR). We selected *Bmp4*, *Fgf10* and *Nkx2-1* (*Ttf-1*) as distal marker genes and *Sox2* and *Scgb1a1* (*CC10*) as proximal marker genes. The critical growth factors involved in branching morphogenesis (*Bmp4*, *Fgf10*, *Shh*) were examined at 6 hours and 24 h. As anticipated, IQ1 significantly decreased the expression of the distal genes, *Bmp4*, *Fgf10* and *Nkx2-1* (Figure 4A). In addition, whole mount *in situ* hybridization confirmed the decrease of the distal genes *Bmp4* and *Nkx2-1* expressed in the distal epithelium (Figure 4B). At same time, IQ1 significantly increased the expression of the proximal genes, *Sox2* and *Scgb1a1* (Figure 4A). However, IQ1 did not decrease the expression of the widely expressed lung epithelial marker *Shh*, suggesting that IQ1 did not disrupt the overall growth of the lung epithelium (Figure 4A). Our results indicate that IQ1 decreased the expression of distal genes and increased the expression of proximal genes in the lung explants consistent with the hypothesis that inhibition of the β-catenin/p300 interaction proximalizes lung epithelium.

### Proximalizations of the embryonic lung selective via inhibition of the β-catenin/p300 interaction or β-catenin deletion are mechanistically distinct

The proximalization resulting from IQ1 inhibition of the β-catenin/p300 interaction phenotypically resembles those seen utilizing genetic deletion of β-catenin [6,14]. Therefore, we decided to compare in detail the mechanisms involved in the pharmacologic inhibition of the β-catenin/p300 interaction to the genetic deletion of β-catenin. β-catenin interacts with many additional transcription partners beyond p300 and CBP. Amongst these, the TCF/LEF family of transcription factors has been most extensively investigated, as consensus LEF1/TCF-DNA binding motifs are a hallmark of canonical Wnt target genes as well as commonly used Wnt signaling reporters. TOPGAL transgenic mice express β-galactosidase under the control of canonical Wnt TCF/β-catenin transcription. To evaluate the effects of specific inhibition of the β-catenin/p300 interaction on Wnt signaling in mouse embryonic lung, we treated lung explants from TOPGAL reporter mice with IQ1. In the vehicle control treated mice, we observed activated Wnt/TCF/β-catenin dependent transcription as judged by β-galactosidase activity at the bronchial epithelium of the proximal airway (Figure 3A, DMSO, arrow). Selective inhibition of the β-catenin/p300 interaction with IQ1 did not decrease the β-galactosidase activity in the proximal airways (Figure 3A, IQ1, arrow), whereas ICG-001, a known inhibitor of β-catenin/CBP interaction, decreased the proximal β-galactosidase activity (Figure 3A, ICG-001, arrow). These results demonstrate that whereas genetic deletion of β-catenin decreases overall Wnt/TCF/β-catenin dependent signaling, on the contrary, selective inhibition of the β-catenin/p300 interaction by IQ1 maintains rather than decreases Wnt/β-catenin/TCF dependent reporter activity in the proximal airways. Furthermore, selective inhibition of the β-catenin/CBP interaction decreases proximal but not distal Wnt/β-catenin/TCF dependent activity without apparently affecting normal lung development *ex vivo*.

β-catenin is a multifunctional protein that beyond its role as a transcriptional activator of Wnt signaling, is also an important protein component of adherens junction, which are important for epithelial cell-cell adhesion. To test if the inhibition of the β-catenin/p300 interaction affected the expression and localization of β-catenin in the lung epithelium, we next examined β-catenin by immunohistochemistry. We found β-catenin widely expressed in both the epithelium and mesenchyme of the control treated lungs (Figure 3B). Localization of β-catenin at the epithelial cell membrane (Figure 3B, arrows) is consistent with its important role in cell-cell adhesion in the lung epithelium. However, inhibition of the β-catenin/p300 interaction by IQ1 did not change the expression or localization of β-catenin whereas deletion of β-catenin completely eliminates it.

Genetic deletion of β-catenin also causes a disruption in angiogenesis of peripheral vessels [6]. To examine if inhibition of the β-catenin/p300 interaction disrupted angiogenesis of peripheral vessels, we immunostained whole mount lungs with an antibody specific for the endothelial cell marker PECAM-1. Compared to the vehicle treated lungs, we found extensive PECAM-1 staining in the capillary endothelium surrounding the distal tips of the IQ1 treated lungs (Figure 3C). This result suggests that IQ1 did not dramatically disrupt angiogenesis of peripheral capillary vessels whereas genetic deletion of β-catenin does.

Based on these experiments, we suggest that although phenotypically similar, the inhibitions of branching morphogenesis via selective pharmacological inhibition of the β-catenin/p300 interaction or genetic deletion of β-catenin are mechanistically distinct.

### Inhibition of the β-catenin/p300 interaction and branching morphogenesis by IQ1 is reversible

**Figure 5:**
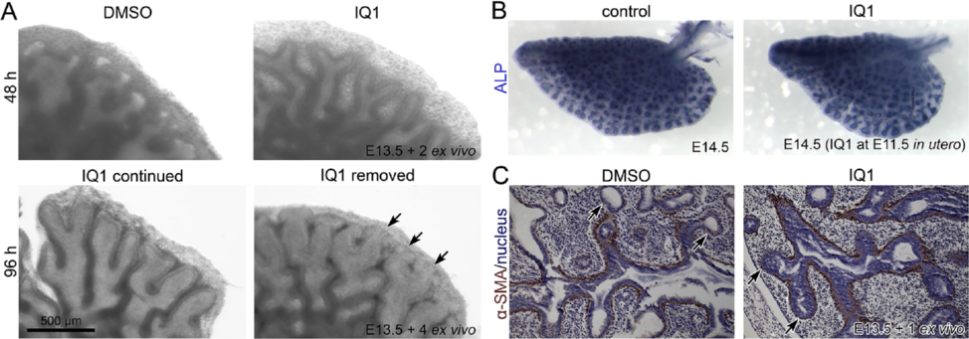
**Branching restarts on removal of IQ1. (A)** E13.5 lung explants were cultured with IQ1 for 48 hours. Subsequently, the IQ1 was removed from the culture media. After two days of the additional culture without IQ1, new branching ends were found at the distal tips (arrows). **(B)** E11.5 embryos were treated with IQ1 *in utero* for one day. The embryos were subsequently dissected at E14.5 after two days of growth in the absence of IQ1. ALP staining of the left lobe shows fine branching equivalent to the control although the lobe is smaller. **(C)** E13.5 lung explants were cultured with either DMSO (control) or IQ1 (10 μM) for 24 hours. Immunohistochemistry of α-SMA shows myofibroblasts surrounding the proximal airway. However, myofibroblasts are absent at the distal tips in both the control and IQ1 treated lung explants (arrows). Nuclei were stained with hematoxylin.

Finally, we addressed the question of whether or not inhibition of branching morphogenesis by IQ1 disruption of the β-catenin/p300 interaction is reversible. To examine this question, we first cultured lung explants with IQ1 for 48 hours and either maintained the treatment with IQ1 or removed the IQ1 from the culture media. After an additional 48 hours of *ex vivo* culture conditions without IQ1, new branching at the distal tips was observed, whereas in the presence of IQ1, distal branching was still absent as anticipated (Figure 5A). Similarly, we treated E12.5 mouse embryos *in utero* for one day and then stopped IQ1 feeding to the pregnant mothers for an additional two days. In the lungs transiently treated with IQ1, we found distal branching tips, essentially equivalent to the untreated controls, although the overall lung lobe was smaller, presumably due to the temporary delay in development (Figure 5B). By immunohistochemistry of alpha smooth muscle actin (α-SMA), a marker of myofibroblasts, we observed myofibroblasts surrounding the proximal airway but not the distal epithelial tips in both the control and IQ1 treated lungs (Figure 5C). The absence of myofibroblasts at the distal tips of the IQ1 treated lungs implies that the distal tips maintain their distal properties and maintain the plasticity to undergo branching morphogenesis upon removal of the inhibitory effects of IQ1. We further speculate that this plasticity, under continuous IQ1 treatment fosters the elongation of the epithelial tubes without branching (Figure 2A). These results suggest that β-catenin/p300 inhibition by IQ1 transiently disrupts lung branching morphogenesis but maintains the plasticity of the epithelial cells at the distal tips for at least 24 hours.

## Discussion

Wnt/β-catenin signaling is critical during multiple stages of lung development [5]. Based upon genetically modified mouse models, Wnt/β-catenin signaling has also been implicated in the regulation of the proximal-distal axis during embryonic lung development. We now provide evidence that interaction between β-catenin and specifically the Kat3 coactivator p300 plays a critical role in proximal-distal axis determination during epithelial lung branching morphogenesis. Inhibition of the β-catenin/p300 interaction, using the previously characterized small molecule inhibitor IQ1 [7] disrupted distal branching of murine lung epithelium *in utero* (Figure 2A) and *ex vivo* (Figure 2D). The phenotype of the IQ1 treated lungs involved proximalization as judged by decreased expression of the distal genes *Bmp4, Nkx2-1* (*Ttf-1*) and *Fgf10* and increased expression of the proximal genes and *Sox2* and *Scgb1a1* (*CC10*) (Figure 4A). *Bmp4* and *Nkx2-1* are expressed in the distal epithelium, whereas *Fgf10* is expressed in the distal mesenchyme [1]. On the other hand, *Sox2* is expressed in the proximal epithelium [15] and *Scgb1a1* is a proximal secretory cell marker, expressed primarily in Clara cells [16]. This proximalization that is induced by specific inhibition of β-catenin/p300 dependent transcription extends our understanding and is fully consistent with previous reports of genetic deletion of β-catenin in the lung epithelium and inhibition of Wnt signaling via *Dkk-1* overexpression inhibiting branching lung morphogenesis [6,14,17]. These earlier investigations in conjunction with our current study, confirm that β-catenin and in particular its interaction with the transcriptional coactivator p300 is critical for proximal-distal determination during lung branching morphogenesis. Our chemical genomic approach that selectively inhibits only a subset of the transcriptional roles of β-catenin, without interfering with its role in adherens complexes at the cell membrane, provides convincing evidence that transcriptional function of β-catenin, not the cell-cell adhesion function is critical for proximal-distal determination. Interestingly, Shikama et al. had previously demonstrated that embryonic mice with even a single allele carrying a defective p300 Histone acetyltransferase (HAT) domain die at birth due to respiratory failure, whereas CBP HAT defective embryos did not demonstrate major lung developmental defects [18]. This is consistent with our results that demonstrate that specific disruption of the β-catenin/p300 dramatically inhibits branching lung morphogenesis, although the phenotypes induced by IQ1 specifically blocking the β-catenin/p300 interaction and knock-in of defective p300 HAT activity do diverge in other respects.

We propose that one of the primary mechanisms by which inhibition of the β-catenin/p300 interaction induces lung proximalization involves the downregulation of *Bmp4* and *Fgf10* expression. *Bmp4* and *Fgf10* are both essential growth factors in lung branching morphogenesis to induce the growth of distal tips [19]. Formation of the distal respiratory epithelium and branching of the proximal airways requires BMP4, which is expressed at the tip of elongating epithelial buds [20]. Inhibition of BMP signaling *in utero* by overexpression of *Noggin* resulted in lung proximalization [20]. *Fgf10* is expressed in the lung mesenchyme and reductions in *Fgf10* expression result in compaction of terminal saccules and neonatal lethality [21,22]. In our study, inhibition of the β-catenin/p300 interaction decreased the expression of both *Bmp4* and *Fgf10* in the lung explants (Figure 4A). The downregulation of *Bmp4* expression was significant already at 6 hours after IQ1 treatment, consistent with BMP4 being a direct Wnt/β-catenin target gene [23]. FGF10 expression although essentially unchanged at 6 hours, was significantly decreased at 24 hours consistent with it being Wnt regulated in the distal lung mesenchyme in an indirect fashion [24]. Our qPCR and *in situ* hybridization data suggest that expression of both *Bmp4* and *Fgf10* is dependent on β-catenin/p300 transcription. Consistent with our results, both *Bmp4* and *Fgf10* have been previously identified as downstream Wnt/β-catenin target genes during proximal-distal patterning in the lung [14,25]. Our result correspondingly indicates that β-catenin/p300 transcription regulates the expression of *Bmp4* and *Fgf10* during lung development. Interestingly, inhibition of the corresponding β-catenin/CBP interaction with ICG-001 did not disrupt branching significantly *ex vivo* (Figure 2E and 3A) and did not affect normal lung development *in utero* (Figure 2B). The significant differences between the effects of the specific small molecule coactivator modulators IQ1 and ICG-001 further demonstrates that Wnt/β-catenin regulated gene transcription is dramatically dependent on its choice amongst the two coactivators, p300 and CBP and that the β-catenin/p300 interaction is essential during lung branching morphogenesis. This result is again fully consistent with the previous results of Shikama et al. [18].

Activation of the Wnt/β-catenin signaling cascade is critical during lung development and has been implicated in the restoration of normal tissue structure and function, as well as remodeling/fibrosis in a number of organs, including the lung, suggesting that this developmental pathway can be reactivated in adult tissues following injury [26,27]. However, exquisite control over reactivation of these developmental pathways is critical, as aberrant activation is associated with lung disease including asthma and fibrosis [28,29]. Therefore, a precise understanding of the role of differential coactivator usage in the Wnt/β-catenin pathway during development and repair after injury is important. In particular, the ability to safely therapeutically modulate coactivator usage has a number of clinical ramifications [30]. We previously demonstrated that administration of the CBP/β-catenin antagonist ICG-001 prevents and reverses established lung fibrosis and dramatically improves animal survival [2]. Similar results have been demonstrated in experimental models of kidney fibrosis [31], and acute lung injury [32]. Impaired branching of the alveolar tree in genetically modified mice leads to respiratory failure resulting from decreased surface for gas exchange. Overall, the lung phenotype bears similarity to respiratory distress syndrome and bronchopulmonary dysplasia, the most common complications of prematurity in humans [33]. Based upon our previous results and the results of our current study, we propose that CBP/catenin antagonists could be safely utilized to treat respiratory distress syndrome and bronchopulmonary dysplasia in infants. In this regard, the recently developed second generation specific Wnt/CBP/catenin antagonist PRI-724 (IC_50_ ~ 150 nM) has proven extremely safe in both Investigational New Drug (IND) enabling toxicology studies and in a Phase Ia clinical trial [34]. In regards to regenerative medicine, *ex vivo* small molecule modulation of stem and progenitors cells offers another avenue to pursue. In that regard, Banerjee et al. demonstrated that human embryonic stem cells (hES cells) directed to alveolar type II (ATII) lung progenitor cells, could be differentiated into an alveolar type I (ATI) phenotype following incubation with the CBP/β-catenin antagonist ICG-001 [35]. One week after acute lung injury induced by bleomycin administration, these differentiated hES cells were able to home to the small airways and engraft, with an accompanied marked reduction in collagen deposition and tissue damage. In the future, the potential to construct bioartificial lungs could provide a source for organs for transplantation. Bioartificial lungs would provide a mechanism to circumvent the problems of donor shortage and immunogenicity. Unlike decellularized lung, synthetic bioartificial scaffolds lack the endogenous growth factors that are required to signal the seeded progenitor cells to essentially recapitulate *ex vivo* the lung development process [36]. Small molecule manipulation of signaling pathways may be used to guide proximal–distal patterning of lung progenitor cells seeded on a synthetic bioartificial scaffold.

## Conclusions

Our study provides strong evidence that the interaction between β-catenin and p300 is critically important in proximal-distal axis determination during mouse lung branching morphogenesis. Our results are consistent and add further refinement to multiple previous reports regarding the role of β-catenin on proximal-distal determination in the lung based upon genetic deletion of β-catenin as well as previous reports on the differential roles of the coactivators CBP and p300 during lung development. The chemical genomic approach utilized herein further elucidated and clarified the specific role of the coactivator p300 in the regulation of Wnt/β-catenin driven branching morphogenesis in the lung. This information, along with the ability to safely pharmacologically manipulate differential coactivator usage in the Wnt/β-catenin cascade provides multiple therapeutic opportunities to investigate going forward.

## Abbreviations

PCR: Polymerase chain reaction
DMSO: Dimethyl sulfoxide
NBT: Nitro-blue tetrazolium chloride
BCIP: 5-bromo-4-chloro-3’-indolyphosphate p-toluidine salt
qPCR: Quantitative polymerase chain reaction
ALP: Alkaline phosphatase
HAT: Histone acetyltransferase
hES cells: Human embryonic stem cells
